# Self Calibrated Wireless Distributed Environmental Sensory Networks

**DOI:** 10.1038/srep24382

**Published:** 2016-04-21

**Authors:** Barak Fishbain, Erick Moreno-Centeno

**Affiliations:** 1 Faculty of Civil & Environmental Engineering, Technion - Israel Institute of Technology, Haifa, 32000, Israel; 2 Department of Industrial & Systems Engineering, Texas A&M University, College Station, TX 77843-3131, USA

## Abstract

Recent advances in sensory and communication technologies have made Wireless Distributed Environmental Sensory Networks (WDESN) technically and economically feasible. WDESNs present an unprecedented tool for studying many environmental processes in a new way. However, the WDESNs’ calibration process is a major obstacle in them becoming the common practice. Here, we present a new, robust and efficient method for aggregating measurements acquired by an *uncalibrated* WDESN, and producing accurate estimates of the observed environmental variable’s true levels rendering the network as self-calibrated. The suggested method presents novelty both in group-decision-making and in environmental sensing as it offers a most valuable tool for distributed environmental monitoring data aggregation. Applying the method on an extensive real-life air-pollution dataset showed markedly more accurate results than the common practice and the state-of-the-art.

## Introduction

### Problem Statement

The increasing availability of sensors and communication technologies have both facilitated^1^,^2^ and catalysed^3^,^4^ the development of Wireless Distributed *Environmental* Sensor Networks (WDESNs) that consist of low-cost Micro Sensing Units (MSUs). WDESNs present an unparalleled means for studying environmental processes such as air-pollution^5^,^6^,^7^,^8^, water quality^9^,^10^, smart cities^11^,^12^ and wildlife ecosystems^13^,^14^. These networks may consist of many sensing nodes and may be deployed over large geographical areas, rendering the calibration process of the nodes as a major obstacle in them becoming the common practice.

Here we present a new, robust and efficient method for aggregating measurements acquired by an *uncalibrated* WDESN, and producing accurate estimates of the observed environmental variable’s true levels. To accomplish that we introduce a new group-decision-making method – consensus aggregation of *incomplete* ratings. The suggested methodology produces accurate results without requiring the MSUs, constituting the WDESN, to be calibrated. Thus, after the aggregation process, the herein proposed methodology renders the network to be self calibrated.

Without loss of generality, let us consider now a WDESN with *K* sensory nodes that measure the same physical phenomenon. The same physical phenomenon can be, naturally measured when the MSUs are collocated^5^,^6^,^7^. Even when the sensors are not collocated measuring the same phenomenon can be achieved when it is uniform in all measuring points^7^. With that, due to the inherent MSUs’ limitations, collocating is currently the common practice^5^,^6^,^7^,^8^. MSU *k* ∈ *K* measures pollutant’s levels, at a given frequency, generating a time series, ***a***^***k***^. The goal then is to find a consensus time series, ***r***, that agrees the most with all the MSUs’ acquired time series, 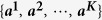. The agreement of ***r*** with each acquired time series, say ***a***^***k***^, is measured by a distance function, *d*(***a***^***k***^, ***r***), that fulfills a set of axioms^15^,^16^,^17^. Examples for *d*() are the *L*_1_ and *L*_2_ norms, and the Kemeny & Snell^16^ and Cook & Kress^15^ axiomatic distances. Thus, given all the MSUs’ acquired time series, 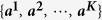, the consensus time series ***r*** is the one that has the minimum sum of distances to all acquired MSUs’ time series:


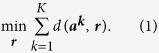


Problem (1) in known as the group-decision-making problem^15^,^16^,^17^,^18^,^19^,^20^,^21^. The group-decision-making problem has been widely-studied and has many applications, such as: voting^18^, jury decisions^19^, consumer opinion aggregation^20^, and project selection^21^. In general terms, the group-decision problem is defined as follows: a group of *K* entities or individuals (referees) collectively evaluate *n* objects. In our context, the evaluations are cardinal evaluations each MSU (referee) assigns to each object (location-time pair) it evaluates. The problem then is to aggregate the referees’ evaluations into a *consensus evaluation* of each and every object. Note that the referee evaluations as well as the consensus evaluation are allowed to contain ties.

For an environmental field campaign that is carried over a time window, *T*, an MSU’s time series, ***a***^***k***^, is considered *complete* if for all time periods *t* ∈ *T*, the MSU gives a valid measurement; otherwise we say that the MSU provides an *incomplete* time series. The latter might happen when MSUs become faulty or switch locations. The latter scenario of sensors switching locations was described by Mead *et al*.^5^, Moltchanov *et al*.^7^ and Lerner *et al*.^8^. Regardless of the incompleteness of the MSUs’ time series, we require that the consensus time series is complete. All previous group-decision work has considered the specific case of complete evaluations^15^,^16^,^17^. Here we introduce and solve the *incomplete* group decision making problem. To this end we (i) introduce a set of natural axioms that must be satisfied by a distance, *d*(), between *incomplete* ratings (time series); (ii) prove the uniqueness and existence of a distance, herein called the *normalized projected Cook-Kress distance* - *d*_*NPCK*_, which satisfies these axioms; and (iii) provide an efficient and practical method for finding the optimal rating (time series) r* for problem (1) when using *d*_*NPCK*_ as the distance function. While we present the new axiomatic distance in the context of WDESNs, it can be used for data fusion in many complete or incomplete group-decision-making application in general and in distributed sensing applications in particular. In our WDESN context, and specifically when presenting the study below, we use the term *time series* when referring to a vector of measurements (provided by an MSU or the consensus time series provided by the aggregation process), while when presenting the methodology, we use the term *rating* in order to emphasize that our proposed methodology is general and applies to aggregating any set of ratings (i.e., vectors consisting of cardinal evaluations).

### Air Quality Wireless Distributed Sensor Networks

Air-Pollution (AP) is known to increase risks for a wide range of diseases, such as respiratory and heart diseases. Recent data indicate that in 2010, 223,000 deaths from lung cancer worldwide resulted from air pollution^22^. This number is expected to grow as studies indicate that in recent years exposure levels have increased worldwide with a significant raise in rapidly industrialising countries with large populations^23^. Studying AP and its impact on health, requires accurate exposure assessments. AP related exposure metrics, typically used in environmental epidemiology studies, are based either on short term sampling^24^ or on pollutant measurements by regulatory standard Air Quality Monitoring (AQM) stations over extended time periods^25^. AQM stations provide accurate measurements but suffer from limited deployment due to their bulkiness, high costs, and their frequent maintenance and calibration requirements. The limited deployment tampers the AQM network’s ability to adequately capture air pollutant spatial concentrations because these concentrations are highly variable. In contrast, intensive sampling campaigns use a large number of AP sensors, deployed at high densities, but are limited to relatively short time periods^22^. Consequently, accurate exposure assessment and the study of AP-health associations are still challenging tasks^26^.

Since AP-MSUs cost significantly less than AQM stations, MSUs can be spread more densely and thus provide data with higher spatial resolution. However, MSUs are error-prone, may become faulty, have limited coherence over time and are inaccurate when compared to AQM stations^5^,^6^,^7^,^8^. Early studies that evaluated MSUs’ capabilities in a controlled lab environment^27^,^28^ stressed the need for a calibration process in order to sustain reliable measurements. Field deployments of such MSUs, measuring ambient *O*_3_ levels by metal-oxide sensors^6^, and measuring *CO*, *NO* and *NO*_2_ by electrochemical^5^ or metal-oxide^29^ probes, have shown that calibration processes applicable for controlled lab environments do not work in the field, when the calibrated data is compared to data collected at a collocated standard AQM station^6^,^7^ (even after an initial field calibration has been applied^6^). Thus, the field calibration process is a critical hurdle that one must overcome, in order to make WDESN a viable tool for AP exposure assessment. Having said that, the suggested method is applicable to many WDESN applications, even though the examples here focus on AP-WDESNs.

## Methods

In this section the set of axioms that a distance metric between *incomplete* ratings must fulfil so that, when using this distance within problem (1), the obtained consensus rating appropriately minimizes the disagreement of the judges (MSUs’ measurements in our context) is presented. In doing so, our aim is to have a distance that is appropriate to aggregate the measurements obtained by uncalibrated MSUs.

Each sensor presents two types of errors - normal measurement error and calibration error. The former is typically considered to be additive, normally distributed with zero mean and constant standard deviation over time^30^,^31^,^32^. The later is assumed to be independent from other sensors’ errors; and roughly stable throughout the measurement collection process/timeframe. The mean calibration error is assumed to be zero, though no assumption is made on the shape of the distribution. Finally the calibration error is considered to be additive. In case of multiplicative error, the algorithm can deal with this in two ways: (1) The algorithm can be applied as is and still obtain meaningful results to the extent that the multiplicative error is significantly smaller w.r.t. the readings themselves. (2) Otherwise, one can take the logarithm of each measurement and apply the (unchanged) algorithm to this re-scaled data because this data re-scaling effectively transforms a multiplicative error to an additive error (since log(*ab*) = log(*a*) + log(*b*)).

The essence of the proposed method is that, due to the calibration error, given any one of the MSU’s the difference between any pair of its measurements is significantly more reliable than the absolute value of the measurements themselves. As such, these differences among the same MSU measurements will be the focus of the following definitions, the axioms proposed, and the resulting distance. Specifically, the main aim of our method is to extract as much information as possible from the reliable measurement differences, and then, with that information in hand, solve the bias problem as a second step (this will be most evident in the solution procedure described at the end of this section).

### Notation and Definitions

Let us consider two arbitrary incomplete ratings, ***a*** and ***b***, in a universe *V* of *n* objects; each rating evaluating the objects in 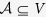 and 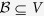, respectively. Hereafter, we represent a rating as a vector of the form ***a*** = (*a*_1_, *a*_2_, …, *a*_*n*_), where *a*_*i*_ is the score (a cardinal evaluation) of object *i* if object *i* is evaluated in ***a*** and *a*_*i*_ is undefined otherwise. We also assume without loss of generality that the possible scores are contained in some pre-specified interval 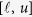; this assumption is without loss of generality, as the MSU’s have a limited measurement range 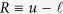. Given two arbitrary incomplete ratings ***a*** and ***b***, the following concepts are defined.

**Definition 1.** *Given a rating **a** and a subset S of the object universe V*, *the projection of **a** on S*, *denoted as **a***|_*S*_, *is the rating of the objects in S that preserves the scores specified by **a** to the objects in S* (*similarly, the objects in S that were not evaluated by **a***, *will remain un-evaluated in **a***|_*S*_).

The following three definitions are natural extensions for incomplete ratings of the corresponding definitions given for complete ratings by Cook & Kress^15^:

**Definition 2.** *Rating **a** is said to be adjacent to rating **b** if* |(*a*_*i*_ − *a*_*j*_) − (*b*_*i*_ − *b*_*j*_)| ≤ 1 *for every pair of objects i and j in the set* 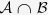. *That is*, *if for every pair of objects their score difference in rating **a** is either the same as in rating **b** or differs by exactly one unit*.

**Definition 3.** *Rating **a** is said to be adjacent of degree k to rating **b** if **a** is adjacent to **b** and*


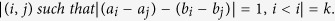


*That is, if the number of object pairs in the set* 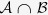 *for which their score difference differs by one unit is k*.

**Definition 4.** *Rating **b** is between ratings **a** and **c** if, for every pair of objects i and j in the set* 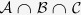, *either a*_*i*_ − *a*_*j*_ ≤ *b*_*i*_ − *b*_*j*_ ≤ *c*_*i*_ − *c*_*j*_ *or a*_*i*_ − *a*_*j*_ ≥ *b*_*i*_ − *b*_*j*_ ≥ *c*_*i*_ − *c*_*j*_.

**Definition 5.** *Ratings **a** and **b** are opposite ratings on* 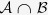, *if* (1) ***a** rates* 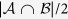 *objects* (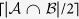 *if* 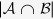 *is odd) with the highest possible score*, *while **b** rates those objects with the lowest possible score*, *and* (2) ***a** rates the remaining objects with the lowest possible score*, *while **b** rates those remaining objects with the highest possible score*. (*Intuitively*, *two opposite ratings are ratings in total disagreement when considering only the objects evaluated by both ratings*).

### Axioms

The objective is to design a distance such that, when used within problem (1), the obtained consensus rating minimizes the disagreement of the judges (uncalibrated MSUs in our context). Following is a set of axioms that a distance metric between *incomplete* ratings must satisfy so that our objective is achieved. Remark: when designing these axioms, we have in mind that, (i) given any MSU, the difference between any pair of its measurements is significantly more reliable than the absolute value of its measurements themselves; and (ii) an MSU providing a large amount of measurements for a particular location is not necessarily more reliable/accurate than an MSU providing a comparatively smaller amount of measurements.

**Axiom 1** (Relevance) 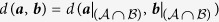


**Axiom 2** (Nonnegativity) *d*(***a***, ***b***) ≥ 0

**Axiom 3** (Commutativity) *d*(***a***, ***b***) = *d*(***b***, ***a***)

**Axiom 4** (Incomplete Ratings Triangular Inequality) 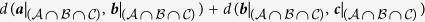 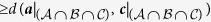 , and equality holds if and only if 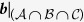 is between 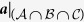 and 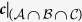


**Axiom 5** (Proportionality) The distance between any two adjacent ratings is proportional to the degree of adjacency

**Axiom 6** (Normalization) *d*(***a***, ***b***) ≤ 1; and *d*(***a***, ***b***) = 1 if and only if 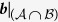 and 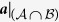 are opposite ratings

It is important to note that Axioms 2 to 4 for incomplete ratings are natural extensions of Cook & Kress’ non-negativity, commutativity and triangular inequality axioms for complete ratings. Indeed, these two sets of axioms are identical when restricted to complete ratings. Similarly, Axioms 5 and 6 are a natural extension of Cook & Kress’ proportionality axiom; the only minor difference is that Cook & Kress’ axiom fixes the proportionality constant to ‘1’; while our normalization Axiom 6 (as shown later), sets the proportionality constant to the reciprocal of 
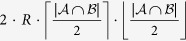
. This minor difference is critical in the context of aggregating incomplete ratings. Specifically, normalization guarantees that when solving problem (1) all of the incomplete ratings are given the same importance regardless of the number of objects that each evaluated—this is critical since larger amounts of data/measurements does not necessarily mean higher accuracy.

### Normalized Projected Cook-Kress Distance

Having this set of axioms, we now define the Normalized Projected Cook-Kress (*d*_*NPCK*_) distance. To do this we use the Cook & Kress distance (*d*_*CK*_) for complete ratings. From our above discussion, it follows that *d*_*CK*_ satisfies the nonnegativity, commutativity, triangular inequality, and proportionality axioms (i.e. Axioms 2 to 5) when focusing on complete ratings^15^. Cook & Kress’ distance is


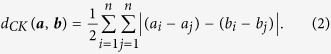


The Normalized Projected Cook-Kress (NPCK) distance is given by:


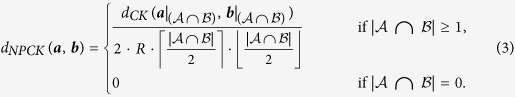


The following sequence of results will allow us to prove that the *d*_*NPCK*_ distance is the unique distance satisfying Axioms 1 to 6 simultaneously.

**Lemma 6.** *Given a set V of n objects and a rating interval* 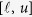 (*evaluation range is* 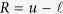), *the maximum Cook & Kress distance*, *d*_*CK*_(•, •), *between any two (complete) ratings is* 
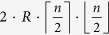
*. Moreover*, *this maximum distance is attained by any two opposite ratings*.

*Proof Sketch*. *The lemma can be restated as follows: “Any pair of opposite (complete) ratings is a global maximizer of problem (4) with an optimal objective value of* 
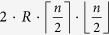
.”


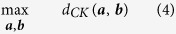


Using Eq. (2) and since ***a*** and ***b*** must be complete ratings within the interval 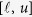, problem (4) can be re-written as


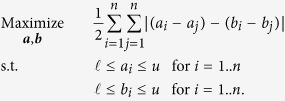


It can be shown that, when ***a*** and ***b*** are assigned values so that they are opposite ratings, (i) one obtains a local maximum of the problem (all feasible directions are non-increasing), and (ii) the objective value of such assignment is equal to 
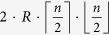
. Since the above optimization problem is convex, every local maximum is a global maximum and thus the result follows.          ◻

**Lemma 7**. *The NPCK distance satisfies Axioms* *1* *to* *6*.

*Proof*. *The NPCK distance*, *d*_*NPCK*_, *satisfies Axiom 1 directly from its definition*, Eq. (3). *The fact that* *d*_*NPCK*_ *satisfies Axioms 2, 3 and 5 follows from* Eq. (3) *and* *d*_*CK*_ *satisfying Cook & Kress’ non-negativity, commutativity and proportionality axioms*.

*d*_*NPCK*_ satisfying Axiom 4 follows from Eq. (3); the fact that 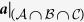, 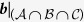, and 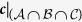 are complete ratings on the set 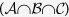; and *d*_*CK*_ satisfying Cook & Kress’ triangular inequality axiom.

Finally, from Lemma 6 and Eq. (3), *d*_*NPCK*_ satisfies Axiom 6.          ◻

**Corollary 8.** *Axioms* 1 *to* 6 *are consistent*.

**Theorem 9.** *The d*_*NPCK*_ *distance is the unique distance satisfying Axioms* 1 *to* 6 *simultaneously*.

*Proof*. *The fact that* *d*_*NPCK*_ *satisfies axioms 1 to 6 was established in Lemma 7. Thus, we only need to show that no other distance satisfies axioms 1 to 6 simultaneously*. *Let* *d* *be a generic distance satisfying axioms 1 to 6. We prove the theorem by showing that, for any two ratings* ***a*** *and* ***b***, *d*(***a***, ***b***) = *d*_*NPCK*_(***a***, ***b***). *We divide our analysis in the following two cases:*

**Case 1:** Both ***a*** and ***b*** are complete ratings (i.e., 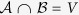).

For complete ratings, Axiom 1 is a tautology and, as argued above, axioms 2 to 5 are identical to all of Cook & Kress’ axioms except for the proportionality constant. Therefore, for complete ratings, Axioms 2 to 5 uniquely determine *d*_*CK*_ except for a proportionality constant. Consequently, since *d*(***a***, ***b***) satisfies Axioms 1 to 5 we conclude that, for complete ratings,


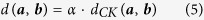


for some constant *α* that may depend only on |*V*| and *R*.

Also, from Eq. (3) and since 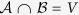, we have that


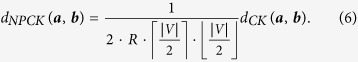


In view of eqs (5) and (6), in order to conclude that *d*(***a***, ***b***) = *d*_*NPCK*_(***a***, ***b***) for complete ratings, we only need to prove that


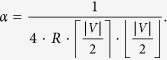


This result follows since both *d*(***a***, ***b***) and *d*_*NPCK*_(***a***, ***b***) attain their extreme values (zero and one) at lots of rating pairs. Specifically, given *any* two opposite ratings, say ***a***′ and ***b***′, axiom 6 stipulates that *d*(***a***′, ***b***′) = *d*_*NPCK*_(***a***′, ***b***′) = 1. Similarly, given *any* rating, say ***a***′, eqs (5) and (6) and the definition of *d*_*CK*_ (Eq. (2)) imply that *d*(***a***′, ***a***′) = *d*_*NPCK*_(***a***′, ***a***′) = 0.

**Case 2:** At least one of ***a*** or ***b*** is an incomplete rating.

The following equalities show that *d*(***a***, ***b***) = *d*_*NPCK*_(***a***, ***b***) for any two ratings ***a*** and ***b*** under the assumptions of this case:


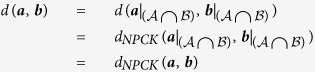


The first and last equalities follow from Axiom 1, while the second equality follows from our analysis of case 1 and the fact that 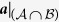 and 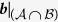 are complete ratings over the set 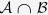.       ◻

### Finding the Consensus Rating

The NPCK distance generalizes the distance between complete ratings proposed by Cook & Kress^15^. Hochbaum and Levin^33^ showed that this complete-rating aggregation problem is a special case of their own separation-deviation model, and thus efficiently solvable. Similarly, given all the MSUs’ acquired time series, 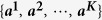, the incomplete-rating aggregation problem (Eq. (1)) using the NPCK distance is a special case of the separation-deviation problem and can be reformulated as:


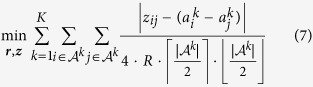



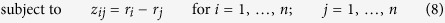



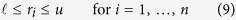


Problem (7) is a special case of the convex dual of the minimum cost network flow problem, and thus it can be solved in 
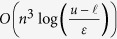
 time^34^, where *n* is the number of objects (in our context, number of time points when the measurements were taken), and *ε* is the desired accuracy.

Finally, recall that our aim when designing the distance function was to extract as much information as possible from the reliable measurement differences (in contrast to the unreliable absolute measurements). Indeed in Problem (7) the bias of each MSU is completely ignored; specifically, Problem (7) can be interpreted as finding the vector ***r***, whose pairwise differences, ***z***, are as close as possible to the given MSU’s pairwise measurement differences 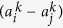. This is precisely what we aimed for because the MSU’s are uncalibrated and thus the MSU’s pairwise measurement differences are significantly more reliable than the absolute values of the measurements. Now, note that given any optimal solution to Problem (7), say r*, the vector ***r***′ = r* + *c*, for any given scalar constant *c*, has exactly the same pairwise differences, z*, and thus is also an optimal solution to Problem (7). As such, the last step of our MSU aggregation method, is to calibrate our aggregated/consensus “measurements”, r*. In particular, we need to find the best calibration constant, *c*, to calibrate our consensus measurement vector r* (keeping fixed all of its pairwise differences, z*). This is achieved by solving the problem


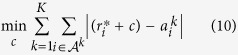



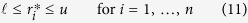


We note that Problem (10) is efficiently solvable by a simple binary search procedure over *c*. Indeed it can be shown that the objective functions of problems (7) and (10) can be combined in a single objective function by adding them and multiplying the objective function of Problem (7) by a large constant so that it is lexicographically more important than that of Problem (10). Moreover, the resulting combined optimization problem would still be a special case of the separation deviation problem and thus efficiently solvable.

The end result after the integration process (i.e., solving problems (7) and (10)) is a set of aggregated and calibrated measurements 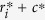 for *i* = 1, …, *n* from the WDESN.

## Results

### Experimental Setup

The suggested methodology was applied on six longitudinal air-quality field campaigns. All campaigns were held in the city of Haifa, located at the eastern Mediterranean sea at the north of Israel (~595,000 residents in Haifa and its satellite cities). The city is built on and around the Carmel Ridge, from the shore at the foot of the ridge to its top at ~400 [m] above sea level (a.s.l.). Ambient levels of nitrogen dioxide (*NO*_2_) and ozone (*O*_3_) were acquired by metal-oxide (MO) sensors (Elm, by Perkin-Elmer, USA) and nitrogen oxide (*NO*) and carbon monoxide (*CO*) by electrochemical (EC) based MSUs (model AQMesh, produced by GeoTech, UK). EC and MO are currently the only available technologies for measuring gaseous pollutants with MSUs^5^,^6^,^7^,^8^,^9^. In all campaigns an array of MSUs was placed next to a standard AQM station. Three different AQM stations were involved in the research reporting pollutants ambient levels. “Igud” AQM station, which is located at the Haifa District Municipalities Association for the Environment (HDMAE) headquarters, at the heart of the Haifa bay heavy industrial area (~30 [m] a.s.l., Lat:32.789379, Lon:35.040452); “Tel-Hai” HDMAE AQM station, located at a Haifa residential neighbourhood on the Carmel Ridge (~200 [m] a.s.l., Lat:32.787293, Lon:35.021072); and “Atzmaut” AQM station (~8 [m] a.s.l., Lat:32.81644, Lon:35.00167), which is operated by the Israeli ministry of environmental protection and is dedicated to measure transportation related pollutants, i.e., *NO* and *CO*. The campaigns details are reported in Table 1.

**Figure 1:**
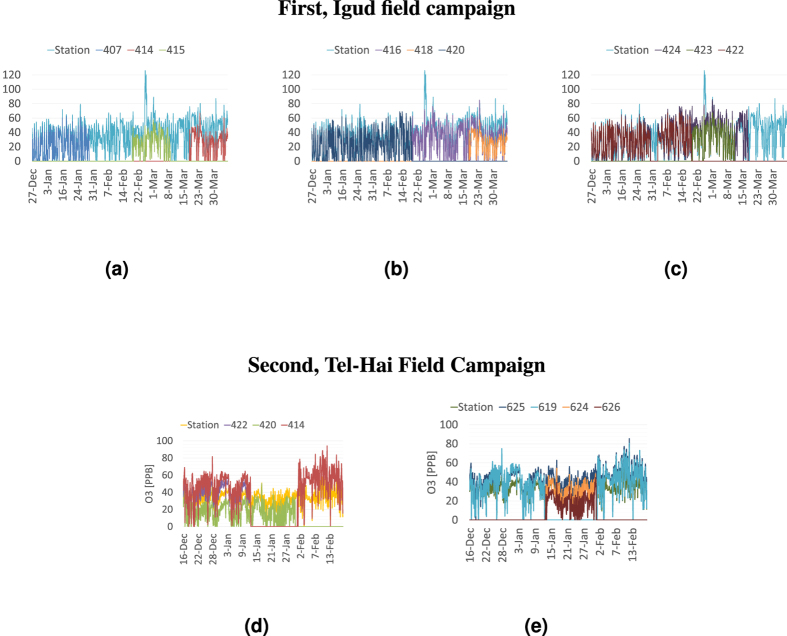
*O*_3_ time series acquired by an AQM and collocated MSUs. First, Igud field campaign, Figures (**a**–**c**), (**a**) 407, 414, 415 and AQM; (**b**) 416, 418, 420 and AQM; (**c**) 422, 423, 424 and AQM. Second, Tel-Hai Field Campaign, Figures (**d**,**e**). (**d**) 414, 420, 422 and AQM; (**e**) 619, 624, 625, 626 and AQM.

To illustrate the acquired data, Fig. 1 depicts ozone time series acquired in Igud and Tel-Hai in the first and second campaigns. The AQMs’ complete data is plotted alongside the incomplete time series acquired by 9 (ID numbers 407, 414, 415, 416, 418, 420, 422, 423 & 424) and 7 (numbers 414, 420, 422, 619, 624, 625 & 626) collocated MSUs at the Igud and Tel-Hai stations respectively. Note that, at different dates, MSUs 414, 420, and 422 switched locations between Igud and Tel-Hai—irrespectively of the common MSUs, the data for each campaign was aggregated independently of that of the other campaign.

### Consensus time series Evaluation

For each campaign and each pollutant three consensus time series were obtained by solving problem (1) with the three different distance metrics—*L*_1_, *L*_2_ and *d*_*NPCK*_. Table 2 presents the coefficients of determination, *R*^2^, between each of the three consensuses and the AQM measurements. This table also details the confidence intervals (CI) of the standard deviations of consensus measurements grouped by the AQM measurements. Thus, how disperse are the consensus measurements for each time the AQM reported the same value. The CI is calculated as the standard error multiplied by the critical two-tailed value of z for *α* = 0.05^35^. Note that the consensus time series obtained when using the NPCK distance when solving problem (1) present, most of the times, higher *R*^2^ and lower CI values as compared to those obtained when using either the *L*_1_ and *L*_2_ distances. Specifically, the NPCK has shown higher *R*^2^ and lower CI for both *NO*_2_ campaigns and for three, out of the four *O*_3_ campaigns. In addition, when the NPCK does not present the best results, it is not far behind presenting almost the same score. Therefore, we conclude that the consensus measurements/time series obtained when using *d*_*NPCK*_, is the best fit for estimating the real AQM measurements/time series based on the consensus of all MSUs.

**Figure 2:**
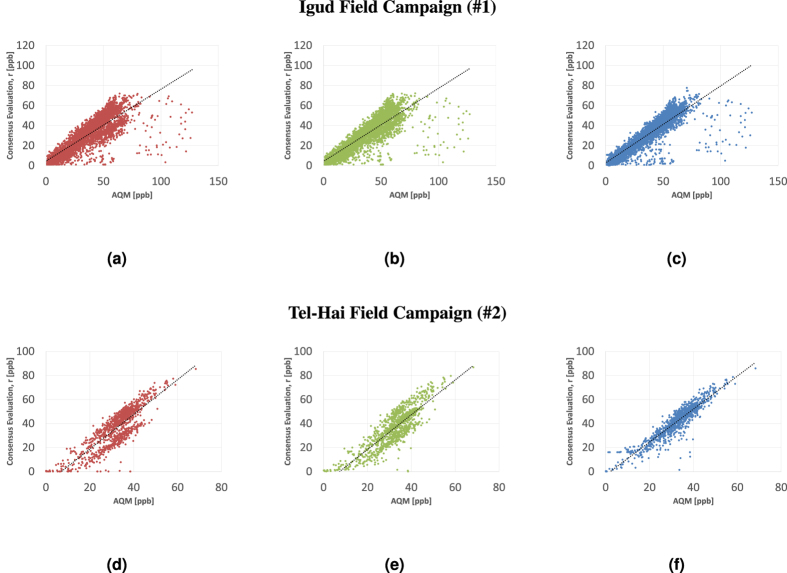
Consensus time series Vs. AQM measurements (Metal-Oxide MSUs). Igud Field Campaign, Figures (**a**–**c**), Tel-Hai Field Campaign, Figures (**d**–**f**). *L*_1_ (**a**,**c**); *L*_2_ (**b**,**d**); and *NPCK* (**c**,**f**).

To illustrate the notions above visually, Fig. 2 plots three consensus time series against the AQM time series obtained for first two campaigns. Each point in the graphs corresponds to a specific time, its x-coordinate is the “measurement” of the consensus time series at that time and its y coordinate is the measurement taken by the AQM at that time. For the Igud campaign, comparing Fig. 2a,b with Fig. 2c, it is evident that the linear relation between the AQM measurements and the consensus time series is stronger for the NPCK as the measurements spread around the linear line is smaller; this exact same result holds for the Tel-Hai campaign (as evident when comparing Fig. 2d,e with Fig. 2f). Supporting the quantitive analysis above.

### Robustness Analysis

**Figure 3:**
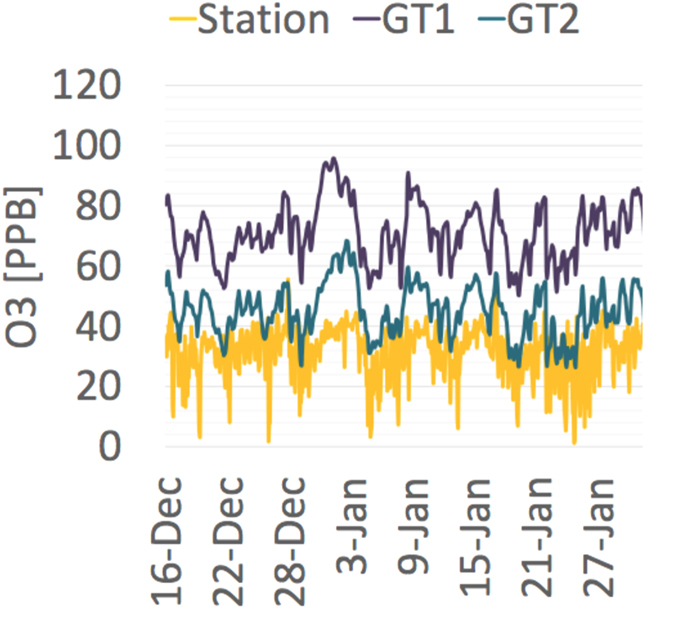
GT135, GT136 and the AQM *O*_3_ measurements throughout campaign 2 (Dec 16^*th*^, 2013 and Feb. 19^*th*^, 2014).

The robustness of the suggested scheme is presented next. For this purpose, two time series (#135 and #136), acquired in the second campaign in conjunction with the data of Fig. 1d,e were added into the aggregation process. These two time series were acquired using EC ozone MSUs (AQMesh of GeoTech, UK). While EC MSUs have been previously used for ozone measurements, this technology suffers heavily from interferences^5^,^6^ and thus, produces measurements that are less accurate than those obtained by using metal-oxide ozone MSUs (which was the type of MSUs used to obtain the data in Fig. 1d,e). Figure 3 presents the Tel-Hai AQM station’s complete time series alongside the incomplete time series measurements acquired from the GT135 and GT136 MSUs. Table 3 depicts the correlation coefficient and the Mean Squared Error (MSE) between the AQM measurements and all sensors that took part in this campaign (see Table 1) and the two added time series (Fig. 3). Note that the last two MSUs added to the process, GT135 and GT136, have a significantly lower correlation and higher MSE then the rest of the MSUs.

**Figure 4:**
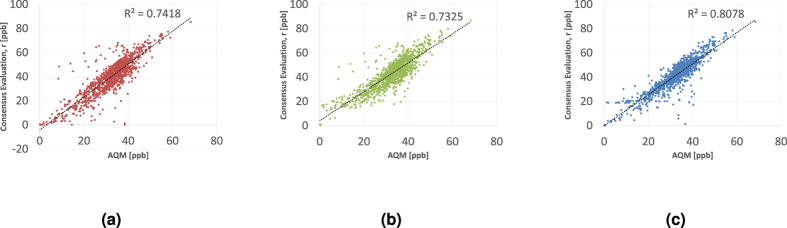
Consensus time series Vs. AQM measurements (Electro-Chemical and Metal-Oxide). (**a**) *L*_1_, (**b**) *L*_2_ and (**c**) *NPCK*.

Figure 4 is analogous to Fig. 2 and plots, against Tel-Hai’s AQM, the consensus “measurements” aggregating both metal-oxide and electro-chemical MSUs when using the *L*_1_, *L*_2_ and *d*_*NPCK*_ metrics within problem (1). Figure 4 also presents the coefficients of determination, *R*^2^, between the three consensuses and the AQM measurements. Similarly to the results when using only metal-oxide MSUs, the correlation coefficient of consensus measurements obtained with *d*_*NPCK*_ is by far the largest one. Therefore, we again conclude that the consensus measurements/time series obtained when using *d*_*NPCK*_, is the best fit for estimating the real AQM measurements/time series based on the consensus of all MSUs.

## Discussion

This paper introduces a scheme for the aggregation of incomplete ratings into a group consensus decision making. The core of the method is the herein-developed axiomatic Normalized Projected Cook-Kress (NPCK) distance. The NPCK distance is derived from a set of axioms any distance between *incomplete* ratings should fulfil so the consensus rating aggregates the given ratings. The consensus rating is the rating that minimizes the sum of all distances from the different ratings. The NPCK approach is an extension of Cook and Kress complete rating aggregation problem, making it suitable to many new applications. An efficient algorithm for finding the consensus rating is also provided.

Wireless Distributed Environmental Sensory Networks (WDESN) have become technically and economically feasible. However, WDESNs may consist of many sensors and thus, the calibration process is a major obstacle. The suggested NPCK distance presents a new, robust and efficient method for aggregating measurements acquired by an uncalibrated, inexpensive and error-prone WDESN, and producing accurate estimates of the observed environmental variable’s true levels. Given a set of collocated Micro Sensing Units (MSUs), the NPCK incomplete ratings scheme is applied, where each measurement (defined by time and location) is considered as a referee evaluation. These time series can be incomplete as sensors might become faulty or shift locations. Based on a set of collocated measurements (in time and in space) a consensus measurement is derived using the NPCK scheme.

The methods have been applied to a wide set of pollutants measurements (i.e., ozone, nitrogen oxide, nitrogen dioxide and carbon monoxide) acquired by all available MSU technologies (metal oxide and electrochemical). When compared to a standard regulatory Air Quality Monitoring (AQM) station, the suggested methodology has shown markedly more accurate results than the common practice and the state-of-the-art, without requiring the Micro Sensing Units (MSUs), constituting the WDESN, to be calibrated, rendering the network to be self calibrated. To achieve this, some assumptions on the error behaviour are made (i.e., additive, zero mean error). While these assumptions are commonly accepted, we have also presented a simple logarithmic data re-scaling technique which enables the method to handle multiplicative errors. Therefore, generalising the suggested scheme even further.

This research has addressed the challenging problem of data aggregation, where only measurements of a single pollutant are aggregated. The interplay between gases in the atmosphere^36^,^37^,^38^, for some gases (e.g., *NO*_2_ and *O*_3_) is known and may allow for the aggregation of data acquired from an heterogeneous set of sensors. Finally, the availability of the code, with the accurate results, present a great potential for making the NCPK the tool of choice for aggregating measurements acquired by uncalibrated WDESNs.

## Additional Information

**How to cite this article**: Fishbain, B. and Moreno-Centeno, E. Self Calibrated Wireless Distributed Environmental Sensory Networks. *Sci. Rep*. **6**, 24382; doi: 10.1038/srep24382 (2016).

## Figures and Tables

**Table 1:** Field Campaigns.

Campaign	Measured Pollutants	Sensor Platform	Sensors #	AQM	Dates
1	*O*_3_	MO	407, 414, 415, 416, 418,420, 422, 423, 424	Igud	27/12/2012–04/04/2013
2	*O*_3_	MO	414, 420, 422, 619, 624,625, 626	Tel-Hai	16/12/2013–19/02/2014
3	*O*_3_, *NO*_2_	MO	414, 422, 624, 626	Tel-Hai	29/04/2014–28/05/2014
4	*O*_3_, *NO*_2_	MO	418, 620, 621	Tel-Hai	09/06/2014–10/07/2014
5	*NO*, *CO*	EC	135, 136, 468	Atzmaut	03/02/2015–26/02/2015
6	*NO*, *CO*	EC	220, 465, 471	Atzmaut	03/02/2015–26/02/2015

**Table 2:** *R*^2^and Std-CI of the consensus time series with respect to the AQM measurements.

Campaign	Pollutant	Count	*R*^2^			Std CI		
			*L*_1_	*L*_2_	*NPCK*	*L*_1_	*L*_2_	*NPCK*
1	*O*_3_	3,439	0.7039	0.7209	**0.8556**	1.2850	1.2883	**0.8410**
2	*O*_3_	1,038	0.7071	0.7922	**0.7922**	**0.9323**	0.9628	0.9357
3	*NO*_2_	1,298	2 · 10^−5^	2 · 10^−5^	2 · 10^−5^	2.6715	2.6896	**2.5783**
	*O*_3_	1,299	0.8730	0.86693	**0.88768**	3.192	**3.0301**	3.1271
4	*NO*_2_	1,420	0.0025	0.0002	**0.0032**	2.999	13.014	**2.9756**
	*O*_3_	1,431	0.7931	**0.8428**	0.8402	**1.4507**	1.6261	1.6478
5	*CO*	2,001	**0.1256**	0.0851	0.1205	3.7435	**3.0432**	3.7111
	*NO*	1,904	0.98088	0.9814	**0.9824**	5.0644	5.3579	**5.0349**
6	*CO*	2,001	0.1446	0.1279	**0.2113**	3.8027	3.9326	**3.7678**
	*CO*	1,766	**0.9462**	0.9428	0.9421	7.9352	7.9078	**7.8121**

**Table 3:** Second Field Campaign MSUs’ characteristics.

	414	420	422	619	624	625	626	135	136
Correlation	0.8710	0.8869	0.9257	0.9147	0.9175	0.9290	0.8903	**0.4779**	**0.5496**
MSE	0.6827	0.5434	0.4180	0.3656	0.3926	0.3243	0.7325	**1.5021**	**0.5862**
